# Utility of Thermal Cross-Linking in Stabilizing Hydrogels with Beta-Tricalcium Phosphate and/or Epigallocatechin Gallate for Use in Bone Regeneration Therapy

**DOI:** 10.3390/polym14010040

**Published:** 2021-12-23

**Authors:** Beiyuan Gao, Yoshitomo Honda, Yoichi Yamada, Tomonari Tanaka, Yoshihiro Takeda, Takayuki Nambu, Shunsuke Baba

**Affiliations:** 1 Department of Implantology, Osaka Dental University, Osaka 573-1121, Japan; gao19940307@gmail.com (B.G.); yamada.yo0925@gmail.com (Y.Y.); takeda-y@cc.osaka-dent.ac.jp (Y.T.); baba-s@cc.osaka-dent.ac.jp (S.B.); 2 Department of Oral Anatomy, Osaka Dental University, Osaka 573-1121, Japan; 3 Department of Biobased Materials Science, Kyoto Institute of Technology, Kyoto 606-8585, Japan; 4 Department of Bacteriology, Osaka Dental University, Osaka 573-1121, Japan; nambu-t@cc.osaka-dent.ac.jp

**Keywords:** cross-link, hydrogel, bone graft materials, β-TCP, bone regeneration therapy

## Abstract

β-tricalcium phosphate (β-TCP) granules are commonly used materials in dentistry or orthopedic surgery. However, further improvements are required to raise the operability and bone-forming ability of β-TCP granules in a clinical setting. Recently, we developed epigallocatechin gallate (EGCG)-modified gelatin sponges as a novel biomaterial for bone regeneration. However, there is no study on using the above material for preparing hydrogel incorporating β-TCP granules. Here, we demonstrate that vacuum heating treatment induced thermal cross-linking in gelatin sponges modified with EGCG and incorporating β-TCP granules (vhEc-GS-β) so that the hydrogels prepared from vhEc-GS-β showed high stability, β-TCP granule retention, operability, and cytocompatibility. Additionally, microcomputed tomography morphometry revealed that the hydrogels from vhEc-GS-β had significantly higher bone-forming ability than β-TCP alone. Tartrate-resistant acid phosphatase staining demonstrated that the number of osteoclasts increased at three weeks in defects treated with the hydrogels from vhEc-GS-β compared with that around β-TCP alone. The overall results indicate that thermal cross-linking treatment for the preparation of sponges (precursor of hydrogels) can be a promising process to enhance the bone-forming ability. This insight should provide a basis for the development of novel materials with good operativity and bone-forming ability for bone regenerative medicine.

## 1. Introduction

β-tricalcium phosphate (β-TCP) was studied by various researchers before 1980 [1,2], but it was not widely used as an osteogenic material for bone disease until this century [3,4,5]. Since its use began, β-TCP has been studied in the form of granules or sponge-like material combined with polymers [6]. In contrast, although the incorporation of calcium phosphate into hydrogels latently impairs the outflow of granules and improves the operability [7,8], scope remains for further investigation to improve the operability and bone regeneration potential.

Gelatin, derived from collagen, is a polymer that has been widely studied in the medical field owing to its biocompatibility, biodegradability, low cost, and inability to induce an immune reaction in vivo [9,10]. In addition to the use of gelatin alone, the polymers had been fused with other components, such as epigallocatechin gallate (EGCG) [11] (an extract of green tea that exhibits anti-inflammatory [11], antibacterial [12], and antioxidant [13] effects), growth factors that control cell proliferation and differentiation [14], and various calcium phosphates [15] including β-TCP [16]. Gelatin for medical use—often obtained from bovine bone and pig skin [17]—has been shown to carry growth factors and promote cell growth [18]. In addition to having high water solubility and the fact that it exhibits the sol–gel reaction at 35 °C, gelatin is useful as a hydrogel component because its mechanical and biochemical properties can be modified by cross-linking [17,18,19]. The cross-linking of gelatin was occasionally performed using chemical cross-linking using condensing agents (1-ethyl-3-[3-dimethylami-nopropyl]-carbodiimide hydrochloride (EDC)/N-hydroxysuccinimide (NHS) [20], glutaraldehyde (GTA) [21], or 4-[4,6-dimethoxy-1,3,5-triazin-2-yl]-4-methylmorpholinium chloride (DMT-MM) [22]), high-energy electron beams [23], γ-irradiation [24], plasma treatment [25], dehydrothermal treatment [26], or treatment with enzymes such as microbial transglutaminase [19,27].

In recent years, we developed EGCG-modified gelatin sponges (Ec-GS) [22], which are chemically cross-linked among gelatin and between gelatin and EGCG by DMT-MM and *N*-methylmorphiline (NMM) in water. These sponges are thermally cross-linked by vacuum heat treatment (vhEc-GS) for further improvement for bone regenerative therapy [26]. vhEc-GS has better bone-forming ability than Ec-GS [26]. Generally, hydrogels do not go through a sponge state during preparation; yet, Ec-GS and vhEc-GS show a hydrogel state when aqueous solution is added, which indicates their potential to be applicable as hydrogel carriers for β-TCP granules. However, the effectiveness of this method has not yet been fully clarified. In this study, we prepared a variety of hydrogels derived from chemically and/or thermally cross-linked gelatin sponges encapsulating β-TCP with or without modification with EGCG to develop exquisite hydrogels for bone regenerative medicine.

## 2. Materials and Methods

### 2.1. Preparation of Sponges (Precursors of Hydrogels)

**Figure 1:**
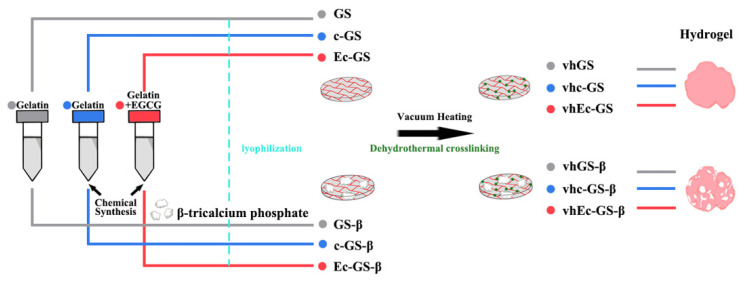
Preparation of sponges (precursor of hydrogels). Process of fabricating sponges.

The following three solutions were prepared to obtain sponges (precursors of the hydrogels): (1) an untreated gelatin solution (gray), (2) a solution with chemically cross-linked gelatin (blue), and (3) a solution with chemically cross-linked gelatin modified with EGCG (left part of Figure 1). In total, six solutions with or without *β*-TCP particles were prepared and freeze-dried to prepare sponges (middle of Figure 1 and Table 1). Then, a part of these sponges was thermally cross-linked using the vacuum heating technique (right part of Figure 1).

Gelatin solution (1%) was prepared by dissolving 100 mg of RM-Gelatin (Jellice Co., Ltd., Sendai, Japan) in 10 mL of ultrapure water. The solution with the chemically cross-linked gelatin and the chemically cross-linked gelatin-modified with EGCG were prepared according to the previously reported methods [22,26]. EGCG (Theaphenon EGCG; Tea Solutions, Hara Office Inc., Tokyo, Japan) was kindly gifted by Dr. Y. Hara. Nine-millimeter holes were prepared in a PTFE sheet (Misumi Group Inc., Tokyo, Japan) and covered at the bottom with a polydimethylsiloxane sheet fabricated with SILPOT 184 (Dow Corning Toray Co., Ltd., Tokyo, Japan). To prepare the sponges, 100 µL of the solution with gelatin, chemically cross-linked gelatin, and chemically cross-linked gelatin modified with EGCG was added to the holes with or without 4 mg of β-TCP (500–1000 μm, CatalyMedic Inc., Chiba, Japan) and placed in a MediFridge at −20 °C (Fukushima Galilei Co., Ltd., Kagoshima, Japan). Subsequently, the frozen material was lyophilized in a DC800 (Yamato Scientific Co., Ltd., Tokyo, Japan) for 24 h to obtain each spongy material. Vacuum heating was performed at 150 °C for 24 h using an AVO-250NS (AS ONE Co., Ltd., Osaka, Japan) and DA-20D (Ulvac kiko. Inc., Kanagawa, Japan) at a gauge pressure of −0.1 MPa. Macroscopic photographs were taken using an EOS 600D digital camera (Canon Inc., Tokyo, Japan). The coating process was carried out using an osmium coater (Vacuum Device Co., Ltd., Ibaraki, Japan) for 3 s, which was followed by observation with an electron microscope. Field emission scanning electron microscopy (FE-SEM) (S-4800; Hitachi Ltd., Tokyo, Japan) was used to confirm the containment of β-TCP granules at 5 kV. Attenuated total reflection-Fourier transform infrared spectroscopy (ATR-FTIR) (IRAffinity-1S; Shimadzu Co., Kyoto, Japan) was used at a wavenumber of 400 to 3900 cm^−1^. X-ray powder diffraction (XRD) (XRD-6000; Shimadzu Co.) was used at angles of 20–40°.

### 2.2. Leakage Test of β-TCP Granules from Hydrogels

The leakage rates of β-TCP from hydrogels were determined using a small test tube with one piece of flake sponge and 1000 µL of ultrapure water. Samples were placed in a refrigerator at 4 °C for 24, 48, and 72 h. After incubation for the prescribed time and elimination of materials without leaked calcium phosphate (CaP), residual CaP were placed in a drying oven (DV 400; Yamato Scientific Co., Ltd.) at 55 °C for 24 h. The scattered β-TCP was weighed using an electronic balance (GR-202; Misumi Group Inc.), and the percentage mass of particles dispersed was calculated using the following formula: β-TCP (mg)/4 × 100%. To confirm the robustness of hydrogels, immunostaining for gelatin was performed using the staining solutions FDV-0035 (10 μg/mL; Funakoshi Co., Ltd., Tokyo, Japan) and Qdot 655 Streptavidin Conjugate (1 μg/mL, Thermo Fisher Scientific Inc., Waltham, MA, USA), following the manufacturers’ instructions. The time required for the hydrogel was tested using a wettability evaluation device (LSE-ME2; Nick Corporation, Saitama, Japan), adding 70 µL water drops for each sponge.

### 2.3. Analysis of Biocompatibility of the Hydrogels

UMR106 (American Type Culture Collection, Manassas, VA, USA) at passage 7 was cultured in 96-well plates (AGC Techno Glass Co. Ltd., Shizuoka, Japan) at a density of 5000 cells per well for 3 d. The culture medium was Dulbecco’s modified Eagle’s medium (DMEM) (MilliporeSigma, Burlington, MA, USA) mixed with 10% fetal bovine serum and 1% antibiotics. Afterward, hydrogels from one disk of vacuum-heated gelatin sponge (vhGS), vacuum-heated chemically synthesized gelatin sponge (vhc-GS), vhEc-GS, vhGS incorporating β-TCP (vhGS-β), vhc-GS incorporating β-TCP (vhc-GS-β), or vhEc-GS incorporating β-TCP (vhEc-GS-β) with DMEM were added to the wells and placed in the incubator. The number of viable cells in the well plates was measured after 24, 48, and 72 h using a Cell Counting Kit-8 (CCK-8; Dojindo Laboratories, Tokyo, Japan). The LIVE/DEAD Viability/Cytotoxicity Kit for Mammalian Cells Protocol was obtained from Thermo Fisher Scientific Inc. UMR106 at passage 7 was seeded on each hydrogel in the amount of 100 μL (20,000 cells/mL) and incubated for 24 h, which was followed by observation using a fluorescent cell imager (Bio-Rad Laboratories Inc., Hercules, CA, USA). The areas occupied by green and red were calculated using ImageJ (Java 1.8.0_172; National Institutes of Health, Bethesda, MD, USA). The following formula was used to obtain dead cells: red area/(red + green area) × 100% (*n* = 3).

### 2.4. Animal Experiment

The experiments were approved by the ethics committee of Osaka Dental University, approval number 21-02017. Eight-week-old *Sprague–Dawley* rats were purchased from Shimizu Laboratory Supplies Co. (Kyoto, Japan) for animal experiments to establish a 9 mm diameter cranial defect model. The defect was treated with 100 µL DMEM (without granules or hydrogels) as the control sham surgery group. Meanwhile, β-TCP granules (12 or 28 mg) or hydrogels containing the same amount of β-TCP granules were implanted as the experimental groups. The unified hydrogels were prepared from seven pieces of sponges with 100 µL medium: vhEc-GS-β(12), a mixture of four pieces of vhEc-GS and three of vhEc-GS-β (4 mg β-TCP), vhEc-GS-β(28), and seven pieces of vhEc-GS-β (4 mg β-TCP). Four rats were used per one group at 3 and 6 w, respectively. The samples were collected by cardiac perfusion fixation after three and six weeks. Ten percent formalin neutral buffer solution was used (Wako Chemicals USA Inc., Richmond, VA, USA) to fix the tissues. Skyscan 1275 (Bruker Co., Billerica, MA, USA) was used for microcomputed tomography (μ-CT) analysis at 80 μA and 70 kV. Samples were decalcified by immersion in decalcifying solution B (Wako Chemicals USA Inc.) for 5 d. After that, tissue sectioning was carried out using the Kawamoto method [28]. Slices were cut at 6 μm thickness using a Leica CM1950 (Leica Biosystems, Wetzlar, Germany). Hematoxylin and eosin (H&E) staining solution was purchased from Muto Pure Chemicals Co., Ltd. (Tokyo, Japan). The tartrate-resistant acid phosphatase (TRAP) staining kit (Wako Chemicals USA Inc.) was used to estimate the appearance of osteoclasts according to the procedure described in the instructions. The amount of osteoclasts was calculated using the following formula: area of purple staining in each picture/total image area × 100% (*n* = 3).

### 2.5. Statistical Analysis

The present study used software Prism 9.0.0 (GraphPad Software, San Diego, CA, USA) for data processing as well as analysis. All statistical analyses were performed using one-way ANOVA Tukey’s multiple comparisons test and presented as mean ± standard deviations. *p* < 0.01 or 0.05 shows significant differences.

## 3. Results

### 3.1. Characterization of Sponges (Precursors of Hydrogels)

**Figure 2:**
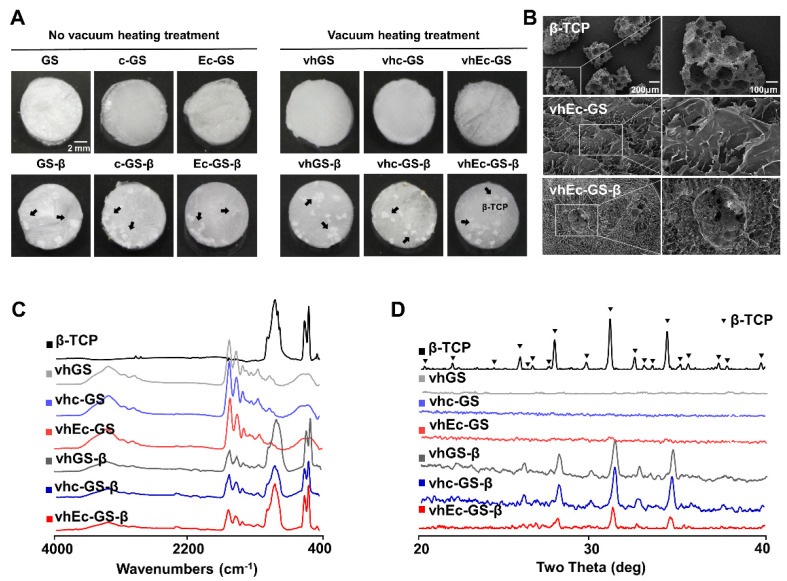
Characterization of sponges (precursor of hydrogels). (**A**) Macroscopic and (**B**) microscopic appearances of the sponges. Arrows: beta-tricalcium phosphate (β-TCP) granules. (**C**) Spectra of attenuated total reflection Fourier transform infrared spectroscopy (ATR-FTIR) and (**D**) X-ray powder diffraction (XRD) on the materials.

Macroscopic observations showed no significant changes in the samples before and after vacuum-heating treatment; FE-SEM observations showed that the porous β-TCP was well encapsulated in all sponges (Figure 2A,B). ATR-FTIR and XRD results confirmed the existence of gelatin and negligible change in the crystalline phase from β-TCP even after the preparation processes (Figure 2C,D). After the characterization of the sponges, the hydrogels were prepared by adding the pure water or medium in the following experiments.

### 3.2. Leakage of β-TCP Granules from Hydrogels

**Figure 3:**
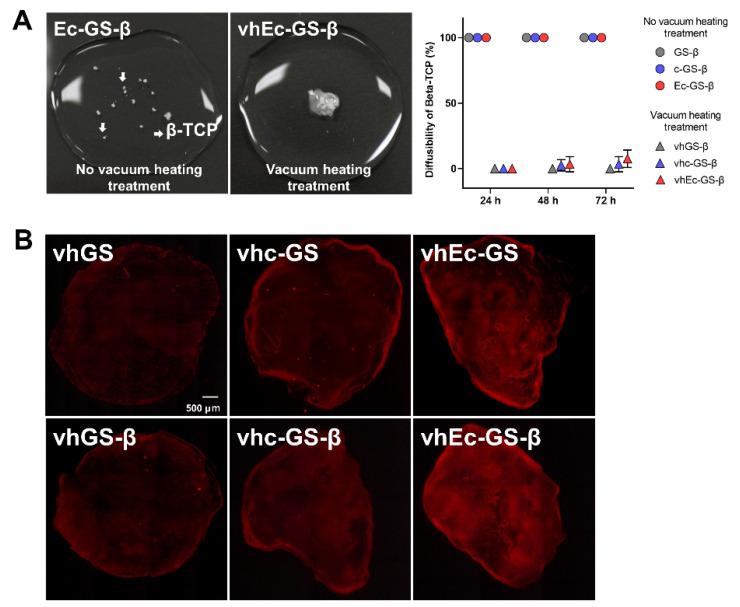
β-TCP leakage and microscopic images of hydrogels. (**A**) Representative macroscopic images of hydrogels and quantitative data after their immersion in water. Arrows: leaked β-TCP granules. (**B**) Microscopic images of hydrogels after gelatin staining.

After the preparation of hydrogels by adding pure water to each sponge, those gels were immersed in aqueous solution and subjected to gentle vibration to estimate the leakage of β-TCP granules. The hydrogels without thermal cross-linking were rapidly disintegrated in the liquid and the granules flowed out, whereas the hydrogels with thermal cross-linking maintained their shape and retained their granules even after 72 h (Figure 3A). Even after gelatin staining with repeated washing, all samples subjected to the thermal cross-linking process retained their shape firmly as hydrogel (Figure 3B). These results suggest that thermal cross-linking enhances the stability of hydrogels and their retention of β-TCP.

### 3.3. Hydrogel Form and Water Absorption Rate

**Figure 4:**
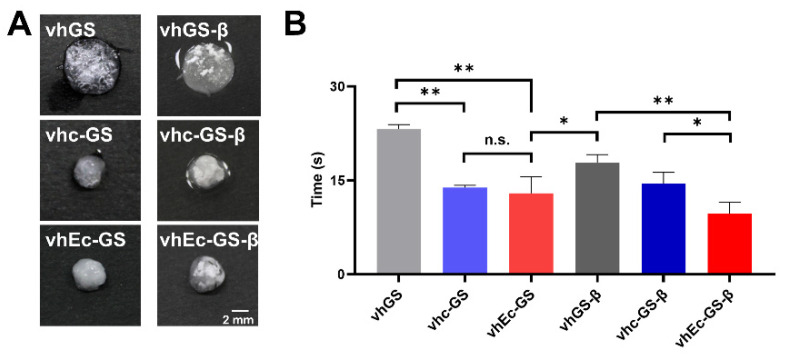
Hydrogel form and water absorption rate. (**A**) Form of hydrogel after the administration of ultrapure water to sponges. (**B**) Water absorption rate. * *p* < 0.05, ** *p* < 0.01. n.s.: not significant.

The forming process of hydrogel occasionally diverges the operability. The water absorption rate of the material is directly related to the preparation time of the specimen [18]. After ultrapure water administration to prepare, the vhc-GS or vhEc-GS and those with β-TCP granules showed great shrinkage, whereas the previous spongy forms of the vhGS and vhGS-β were relatively retained (Figure 4A). The water absorption times of vhEc-GS and vhEc-GS-β were significantly lower than those of vhGS and vhGS-β, respectively (Figure 4B).

### 3.4. In Vitro Biocompatibility Test

**Figure 5:**
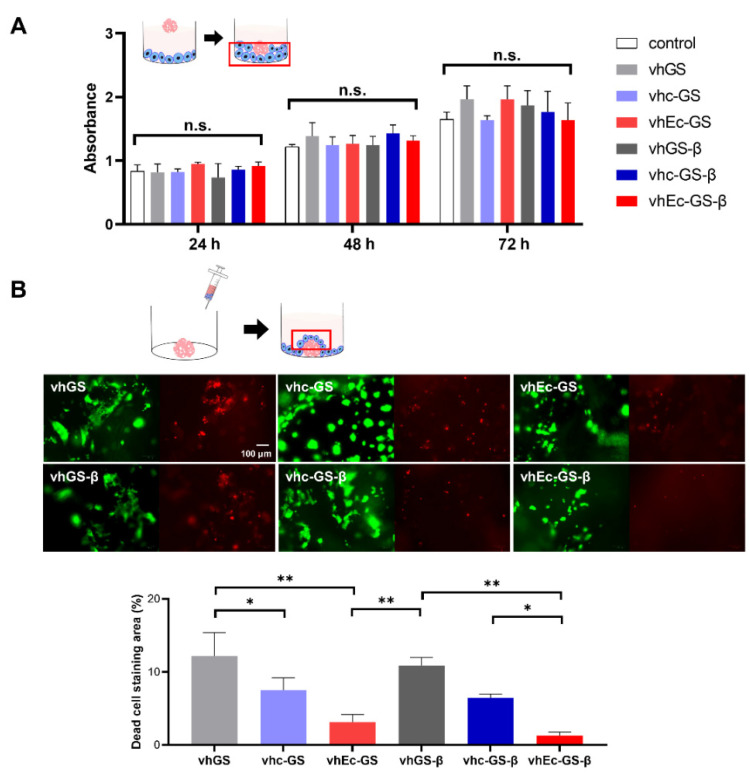
Biocompatibility of hydrogels. (**A**) Cytotoxicity of the hydrogels tested with the CCK-8 assay and osteoblastic cell line UMR106 after 24 to 72 h of incubation (*n* = 3). (**B**) Live or dead staining after 24 h of incubation. Green: live cells on the surface of the hydrogels; red: dead cells (*n* = 3). n.s.: not significant. * *p* < 0.05, ** *p* < 0.01.

Sponges were hydrogelated with DMEM before use in cell culture experiments. Two different methods were used to evaluate the cytotoxicity of hydrogels using the rat osteoblastic cell line UMR106. The results of the CCK-8 test involving cells not in contact with the hydrogels showed negligible cytotoxicity (Figure 5A). In contrast, in live or dead staining for the cells in contact with hydrogels, dead cells could be observed on the hydrogels from vh-GS and vh-GS-β, while there were significantly fewer dead cells on the hydrogels from vhEc-GS and vhEc-GS-β (Figure 5B).

### 3.5. Bone Regeneration and β-TCP Resorption

**Figure 6:**
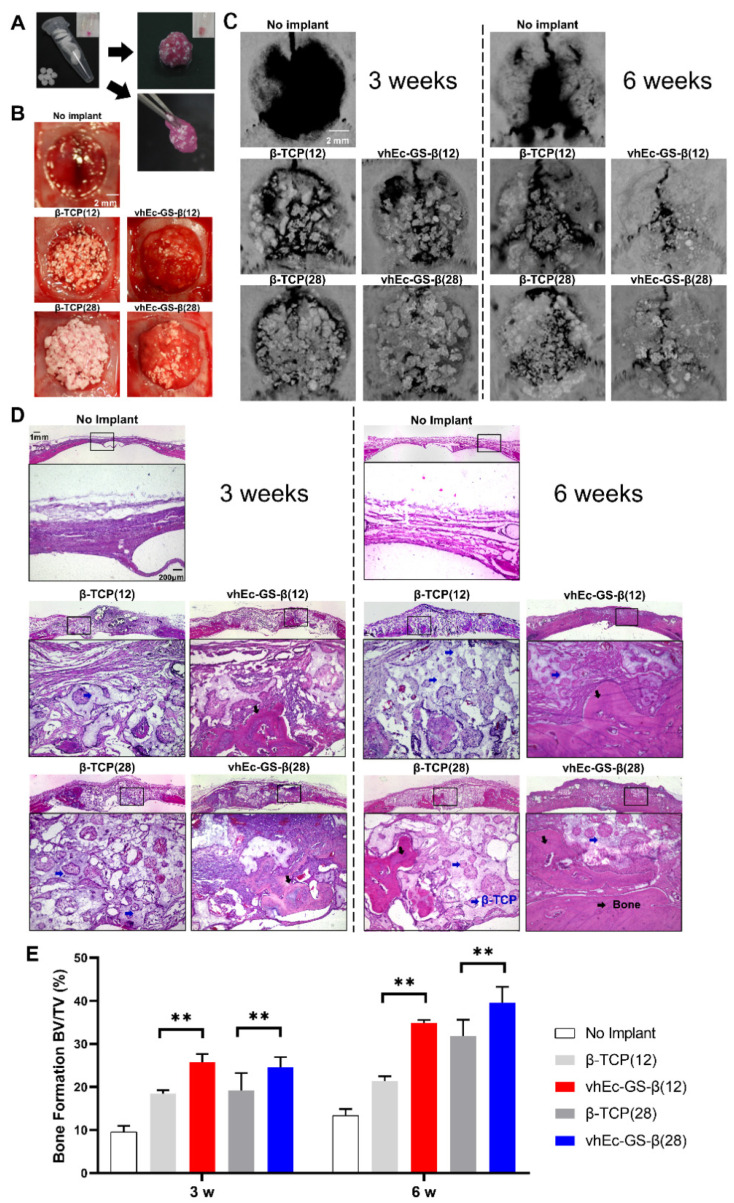
Animal experiments to evaluate bone formation. (**A**) Preparation of the hydrogels: seven pieces of sponges for each hydrogel. (**B**) Macroscopic images of bone defects and those treated with materials right after the surgery (*n* = 4). (**C**) Microcomputed tomography (μ-CT) images of bone defects at three and six weeks. (**D**) Hematoxylin and eosin (H&E) staining of bone defects. (**E**) Quantitative μ-CT analysis after the removal of β-TCP granules. Statistical significance: comparison between granules and hydrogels is presented as follows: ** *p* < 0.01. BV/TV: bone volume per total volume (%).

Given the superiority of vhEc-GS-β in terms of ease of use (compared with granules alone) and cytocompatibility, the bone-forming ability of hydrogels from vhEc-GS-βs and β-TCP granules and the resorption of β-TCP granules were compared using a critical bone defect (9 mm) of rat calvaria (Figure 6). Based on the size of the bone defect, seven pieces of vhEc-GS-β (containing β-TCP 28 mg) or four pieces of vhEc-GS combined with three pieces of vhEc-GS-β (containing β-TCP 12 mg) were mixed with DMEM and integrated to prepare each hydrogel before implantation (Figure 6A,B). The gels were maneuverable and could be grasped with tweezers (Figure 6A). The amount of β-TCP granules was unified respectively at 12 or 28 mg per defect (the numbers in brackets = the weight of β-TCP granules). The μ-CT results showed that the hydrogels from vhEc-GS-β sponges showed an increased opaque image compared to the β-TCP granules alone at three and six weeks after implantation (Figure 6C). H&E staining images ensured that the increased area was attributed to new bone (Figure 6D). Furthermore, μ-CT morphometry analysis after removing the opaque images from the β-TCP granules showed that vhEc-GS-β(12) and β(28) formed significantly greater amounts of bone than did the β-TCP granules alone (Figure 6E).

### 3.6. β-TCP Resorption

**Figure 7:**
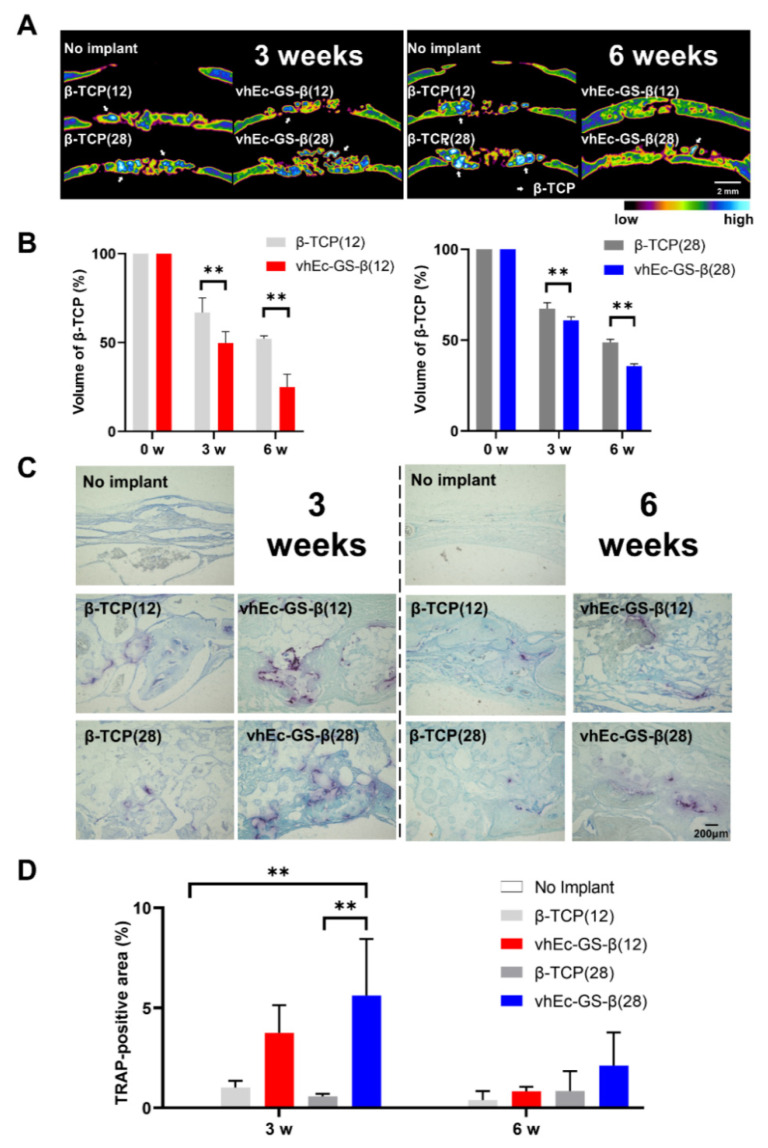
Degradation of β-tricalcium phosphate (β-TCP) in bone defects. (**A**) Lateral view of bone defects at μ-CT analysis. Light and dark blue in bone defects represent residual β-TCP granules. (**B**) Quantification of residual β-TCP granules using μ-CT analysis. (**C**) Tartrate-resistant acid phosphatase (TRAP) staining for osteoclasts. (**D**) TRAP staining area in defects. ** *p* < 0.01.

The lateral view of the bone defect in μ-CT images showed that more light and dark blue granules (indicating β-TCP) could be found in the β-TCP groups than in those in the defects treated with hydrogels from vhEc-GS-β at 6w (Figure 7A). The β-TCP granules in vhEc-GS-β groups were resorbed earlier than those in β-TCP groups (Figure 7A,B). TRAP staining representing osteoclasts revealed that the number of osteoclasts increased at three weeks in the vhEc-GS-β groups compared to those in the β-TCP groups in the case of 28 mg loading (Figure 7C,D).

## 4. Discussion

This study demonstrated that the introduction of a thermal cross-linking process during the preparation of gelatin hydrogels containing EGCG and β-TCP significantly enhanced the retention of β-TCP in the prepared hydrogels. The hydrogels derived from EGCG-modified gelatin sponges containing β-TCP showed greater bone regeneration ability and β-TCP resorption than the use of β-TCP granules alone.

Both great operativity and bone-forming ability are desired properties for bone graft materials used in clinical settings. Complications, such as separation of the graft materials from the graft bed, can lead to bacterial infections and undesirable results [29]. Regarding the application of hydrogels with CaP granules, using robust hydrogels can be a promising strategy to circumvent CaP leakage and overcome these issues. Although various methods have been used to synthesize hydrogels and a wide variety of polymers have been applied, hydrogels are generally synthesized in water and used as they are, or they are suspended in an aqueous solution after synthesis to form hydrogels [30], and hydrogels after sponge formation are scarce. In this study, a solution containing gelatin, chemically cross-linked gelatin, and chemically cross-linked gelatin modified with EGCG was freeze-dried to form a sponge as a precursor to hydrogels, and then thermally cross-linked by vacuum heating, which is followed by the addition of a liquid to obtain each hydrogel (Figure 1). Although there are many processes involved, the freeze-drying process makes it possible to apply physical cross-linking by subsequent vacuum heating. Figure 3 shows that the stability of the thermally cross-linked hydrogel was improved compared to that of non-thermally cross-linked hydrogel, and it was able to retain CaP for a long time.

In previous studies, it was difficult to achieve a strong bonding between gelatin and EGCG when EGCG and gelatin were simply mixed, freeze-dried without aqueous chemical synthesis, and then thermally cross-linked [31]. Based on our previous finding, we used a chemical cross-linking step with aqueous chemical synthesis using DMT-MM and NMM prior to freeze drying to prepare c-GS and Ec-GS. The use of other chemical synthesis conditions, for example, changing of the concentration of DMT-MM and NMM or using organic solvents, could improve the stability of hydrogels [32]. However, in our previous studies, it was difficult to increase the stability of Ec-GS by simply altering the concentrations of DMT-MM and NMM [22]. There are concerns about the cytotoxicity and other environmental effects caused by residual components if organic solvents are used [21,22]. Considering these factors, the used synthesis method, which combines aqueous chemical synthesis to induce chemical cross-linking and vacuum heating to thermal cross-linking, is considered a useful method to produce hydrogels that contain calcium phosphate and have high stability.

Differences in the hydrophilicity of materials are known to affect cell adhesion and cytotoxicity [33]. Meanwhile, the vhEc-GS and vhEc-GS-β absorbed ultrapure water most quickly to form hydrogels (Figure 4B). In vitro cytocompatibility tests showed that hydrogels from vhEc-GS and vhEc-GS-β had the great cell affinity. We had previously shown that heat-cross-linked, untreated gelatin is hydrophobic, whereas vhEc-GS, synthesized in water and heat-cross-linked, was more hydrophilic [34]. Considering these facts, using aqueous chemical synthesis methods before thermal cross-linking for gelatin might partially contribute to enhancing the surface properties of gels, resulting in improved biocompatibility.

Bone defects with φ 9 mm are more difficult to close than other critical sized bone defects in rat experimental models [35]. Indeed, β-TCP could not close the bone defect up to six weeks, whereas the hydrogels from vhEc-GS-β could (Figure 7A). Although the detailed mechanism has not been elucidated here, the following possibilities are considered: (1) the pharmacological action of EGCG, and (2) a scaffolding effect by the stable gelatin structure. Regarding the former mechanism, EGCG is known to promote the differentiation of pluripotent progenitor cells [36]. The implantation of β-TCP produces reactive oxygen species (ROS) [37], which occasionally impair bone formation, while EGCG has been widely recognized as an antioxidant molecule [13,38]. These pharmacological effects may have promoted bone regeneration. Indeed, in vitro experiments have shown a reduction in cytotoxicity (Figure 5B).

Regarding the second mechanism, the presence of a cell scaffold material greatly influences the effect of bone regeneration [39]. Immediately after implantation, β-TCP alone does not have a scaffold to connect the intergranular spaces. β-TCP is known to promote the production of MMPs, which promote the degradation of collagen and gelatin [40,41]. On the other hand, the β-TCP granules in vhEc-GS-β are covered with gelatin, whose stability is enhanced by thermal cross-linking (Figure 3A). Furthermore, EGCG is known to have an inhibitory effect on enzymes such as MMPs [42,43]. In hydrogels from vhEc-GS-β, EGCG may protect the destruction of gelatin, so that the polymer can fill the intergranular spaces and act as a scaffold for cell migration and differentiation, leading to enhanced bone regeneration.

It is well-known that EGCG inhibits osteoclast differentiation and activity [44]. In this study, vhEc-GS-β containing EGCG induced more rapid resorption of the encapsulated β-TCP granules than did β-TCP alone and more osteoclasts, at least in the condition using 28 mg β-TCP. This discrepancy may be due to the different doses of EGCG; EGCG inhibits osteoclast differentiation at >20 μM [15,45], possibly via inhibiting the receptor activator of NF-κB ligand (RANKL) [46], whereas it promotes osteoblast differentiation, such as stem cells, from 1 to 10 μM [47,48]. The dose of EGCG in vhEc-GS was established based on the concentrations that significantly promote osteogenesis from previous studies [26]. Osteoblasts play a key role in generating osteoclasts through RANKL [49]. Thus, the low doses of EGCG adopted in our study are likely to enhance osteoblast differentiation rather than inhibit osteoclast differentiation, thereby promoting the biodegradation of β-TCP granules in hydrogels.

Although hydrogels from vhEc-GS-β showed a better bone-forming ability than β-TCP granules, further careful analysis is necessary before clinical use. For example, the applicability on other gelatins and β-TCPs has not been validated. Gelatin can be available from various animal species, such as fish, marine snail, bovine, caprine, and porcine [50,51]. The physicochemical properties of gelatin are influenced by those origins [50,51]. Different β-TCPs may alter the bone-forming ability of vhEc-GS-β, because the pore size or porosities of calcium phosphate materials are well known to modulate the bone-forming ability [52,53]. The optimal conditions for vacuum heat treatment, as well as the optimal amounts of β-TCP and EGCG, require further investigation. Indeed, our previous study using vhEc-GSs has exhibited the various amount of bone formation according to the dose of EGCG [26]. The previous study reported that the amount of calcium phosphates in gelatin alters the bone formation in the bone defects [54]. Thus, the modification of the above factors may augment the bone-forming ability of vhEc-GS-β or promote the β-TCP resorption rate. In addition, as mentioned above, the mechanism underlying how hydrogels accelerate bone regeneration requires further elucidation. However, the results of this study demonstrate the usefulness of the thermal cross-linking process for the preparation of robust hydrogels retaining β-TCPs. These findings may provide implications for developing novel CaP-incorporated hydrogels applicable to regenerative therapies.

## 5. Conclusions

In this study, we showed that introducing vacuum heating treatment to yield thermal cross-linking for sponges (precursor of hydrogels) increased the stability of the hydrogels, thereby circumventing the leakage of β-TCP granules. There were negligible changes in the sponges at the macroscopic observation or the crystallinity of β-TCPs under the used vacuum heating condition (150 °C for 24 h). vhEc-GS and vhEc-Gs-β significantly absorbed more water than vhGS and vhGS-β. The hydrogel prepared from vhEc-GS-β showed better in vitro cytocompatibility, particularly when the cells were attached to the hydrogels; the hydrogels showed great bone-forming ability than did β-TCP granules in vivo. β-TCP granules encapsulated in the hydrogels from vhEc-GS-β were more rapidly resorbed than were β-TCP granules used alone. These results suggest that thermal cross-linking is a useful method to improve the bone regeneration ability of gelatin hydrogels containing EGCG and β-TCP and may provide an insight into the future formation of new functional hydrogels for bone regenerative medicine.

## Figures and Tables

**Table 1:** Summary of prepared sponges.

Sample Name	Abbreviation	Gelatin (mg)	EGCG (mg)	β-TCP (mg)	Chemical Synthesis	Vacuum Heating
Gelatin sponge	GS	1	0	0	No	No
Chemically synthesized gelatin sponge	c-GS	1	0	0	Yes	No
Chemically synthesized gelatin sponge modified with EGCG	Ec-GS	1	0.0028	0	Yes	No
GS incorporating β-TCP	GS-β	1	0	4	No	No
c-GS incorporating β-TCP	c-GS-β	1	0	4	Yes	No
Ec-GS incorporating β-TCP	Ec-GS-β	1	0.0028	4	Yes	No
Vacuum-heated GS	vhGS	1	0	0	No	Yes
Vacuum-heated c-GS	vhc-GS	1	0	0	Yes	Yes
Vacuum-heated Ec-GS	vhEc-GS	1	0.0028	0	Yes	Yes
Vacuum-heated GS-β	vhGS-β	1	0	4	No	Yes
Vacuum-heated c-GS-β	vhc-GS-β	1	0	4	Yes	Yes
Vacuum-heated Ec-GS-β	vhEc-GS-β	1	0.0028	4	Yes	Yes
